# Ligand-dependent interactions between SR-B1 and S1PR1 in macrophages and atherosclerotic plaques

**DOI:** 10.1016/j.jlr.2024.100541

**Published:** 2024-04-05

**Authors:** Christine Bassila, George E.G. Kluck, Narmadaa Thyagarajan, Kevin M. Chathely, Leticia Gonzalez, Bernardo L. Trigatti

**Affiliations:** Department of Biochemistry and Biomedical Sciences, Thrombosis and Atherosclerosis Research Institute, Hamilton Health Sciences, McMaster University, Hamilton, ON, Canada

**Keywords:** atherosclerosis, high-density lipoprotein, macrophage, scavenger receptor class B type I, sphingosine-1-phosphate, sphingosine-1-phosphate receptor 1

## Abstract

HDLs carry sphingosine-1-phosphate (S1P) and stimulate signaling pathways in different cells including macrophages and endothelial cells, involved in atherosclerotic plaque development. HDL signaling via S1P relies on the HDL receptor scavenger receptor class B, type I (SR-B1) and the sphingosine-1-phosphate receptor 1 (S1PR1), which interact when both are heterologously overexpressed in the HEK293 cell line. In this study, we set out to test if SR-B1 and S1PR1 interacted in primary murine macrophages in culture and atherosclerotic plaques. We used knock-in mice that endogenously expressed S1PR1 tagged with eGFP-(*S1pr1*^*eGFP/eGFP*^ mice), combined with proximity ligation analysis to demonstrate that HDL stimulates the physical interaction between SR-B1 and S1PR1 in primary macrophages, that this is dependent on HDL-associated S1P and can be blocked by an inhibitor of SR-B1's lipid transfer activity or an antagonist of S1PR1. We also demonstrate that a synthetic S1PR1-selective agonist, SEW2871, stimulates the interaction between SR-B1 and S1PR1 and that this was also blocked by an inhibitor of SR-B1’s lipid transport activity. Furthermore, we detected abundant SR-B1/S1PR1 complexes in atherosclerotic plaques of *S1pr1*^*eGFP/eGFP*^ mice that also lacked apolipoprotein E. Treatment of mice with the S1PR1 antagonist, Ex26, for 12 h disrupted the SR-B1-S1PR1 interaction in atherosclerotic plaques. These findings demonstrate that SR-B1 and S1PR1 form ligand-dependent complexes both in cultured primary macrophages and within atherosclerotic plaques in mice and provide mechanistic insight into how SR-B1 and S1PR1 participate in mediating HDL signaling to activate atheroprotective responses in macrophages.

Atherosclerosis is a leading cause of ischemic cardiac, cerebrovascular, and peripheral arterial disease and a major contributor to disease and death globally (1). It is a progressive inflammatory disease initiated with the subendothelial retention, in the intima of artery walls, of apolipoprotein B (apoB)-containing lipoproteins and their subsequent oxidation (2). Oxidized LDL (ox-LDL) activates innate immune cells, particularly monocyte-derived macrophages, which engulf the ox-LDL through their constitutively expressed scavenger receptors on the cell surface (3). Although macrophages effectively clear ox-LDL, it is cytotoxic and can induce macrophage apoptosis as well as necroptosis (4, 5, 6, 7). However, impaired macrophage-mediated efferocytosis (binding and clearance of apoptotic cells) in atherosclerotic plaques results in the persistence of apoptotic cell debris and secondary necrosis, which combined with ongoing necroptosis leads to the release of cell-derived cholesterol, proteases, and cellular-derived damage-associated molecular patterns acting as inflammatory mediators, to drive atherosclerotic plaque development (8, 9).

Numerous epidemiological and preclinical studies have demonstrated that HDL protects against atherosclerosis (10, 11, 12, 13, 14). This has long been attributed to its role in reverse cholesterol transport, a process through which cell cholesterol is taken up by HDL from peripheral cells (including foam cells) and is delivered to the liver for excretion into bile or for recycling back into the bloodstream (15, 16, 17). Hepatic uptake of HDL-cholesterol is mediated by the scavenger receptor class B type I (SR-B1), an HDL receptor that is encoded by the *SCARB1* gene (18, 19). SR-B1 is a 509 amino acid transmembrane glycoprotein, composed of two hydrophobic transmembrane domains and two relatively short cytoplasmic domains at the N and C termini (15, 20). Its extracellular domain contains a hydrophobic channel extending from the HDL-binding site to the cell plasma membrane, allowing the efficient bidirectional movement of cholesterol between bound HDL and cells (21, 22, 23, 24, 25, 26).

HDL also mediates a variety of other antiatherogenic effects including the induction of intracellular signaling pathways in macrophages, endothelial, and smooth muscle cells (5, 6, 27, 28, 29, 30, 31, 32, 33, 34, 35, 36, 37, 38). Many of these effects are mediated by the HDL-associated bioactive lysosphingolipid, sphingosine-1-phosphate (S1P) (28, 30, 31, 32, 33, 37, 39, 40). S1P is generated by the phosphorylation of sphingosine, catalyzed by sphingosine kinases 1 and 2 and irreversibly degraded to phosphoethanolamine and hexadecanal by S1P-lyase (S1PL) (41). Most (65–98%) of the plasma S1P is associated with HDL (42, 43) through binding to a hydrophobic ligand-binding pocket of apo M, associated with a subset of HDL particles (44). S1P mediates a wide spectrum of cellular functions in different types of cells by binding to five related G-protein-coupled receptors, named S1P Receptors (S1PR) 1–5 (45). S1PR1, S1PR2, and S1PR3 are ubiquitously expressed, whereas S1PR4 is primarily expressed in lymphoid tissues and S1PR5 is found almost exclusively in the brain and spleen (46, 47).

We have previously reported that HDL treatment of macrophages in culture stimulates their migration and protects them against apoptosis and necroptosis induced by a variety of stimuli (5, 6, 28, 30). We demonstrated that HDL signaling in macrophages requires SR-B1, which acts as an HDL receptor, an adaptor protein, postsynaptic density protein/drosophila disc-large protein/zonula occludens protein containing 1 (PDZK1), which reportedly binds to the cytoplasmic carboxy terminus of SR-B1 and S1PR1 resulting in Akt 1 phosphorylation (5, 6, 28, 30). On the other hand, HDL-mediated signal transducer and activator of transcription 3 activation in macrophages involves S1PR2 and S1PR3 (35). HDL signaling in endothelial cells also involves SR-B1, both S1PR1 and S1PR3 and HDL-associated S1P (37). Conditional inactivation of *S1pr1* gene expression either in myeloid or endothelial cells increases atherosclerosis in mice (30, 32). Similarly, genetic inactivation of either *Sr-b1* or *Pdzk1* in bone marrow-derived cells increases atherosclerosis in mice, and knockout of either *S1pr1*, *Sr-b1*, or *Pdzk1* in bone marrow-derived/myeloid cells increases apoptosis and necrotic core development within atherosclerotic plaques (5, 30, 48), consistent with the possibility that these three factors participate in the same pathway.

Others have previously reported that when overexpressed in transfected HEK293 cells, SR-B1 and S1PR1 proteins form complexes in the presence of HDL-bound S1P (49). This may explain the functional requirement of both SR-B1 and S1PR1 for HDL signaling that has been reported in macrophages (5, 28, 30) and endothelial cells (37). However, whether SR-B1 and S1PR1 form complexes at physiological levels of endogenous expression in macrophages either in culture or within atherosclerotic plaques has not been tested.

Therefore, we sought to examine if SR-B1 and S1PR1 receptors might interact physically under more physiologically relevant conditions of endogenous expression levels in primary macrophages and in atherosclerotic plaques in mice. We demonstrate that SR-B1 and S1PR1 interact in an HDL-dependent manner in peritoneal macrophages and in cells within atherosclerotic plaques from *ApoE*-deficient mice. We report that this HDL-stimulated interaction depends on HDL-associated S1P, SR-B1’s lipid transfer activity, and S1PR1 activity.

## Materials and methods

### Mice

All procedures involving experimental mice were first approved by the McMaster University Animal Research Ethics Board and were in accordance with the Canadian Council of Animal Care guidelines. Mice were bred and group housed at the David Braley Research Institute animal facility under controlled temperature and light and provided with free access to automatic watering and standard chow diet. All mice were on a C57BL/6J background. *Sr-b1*^*KO/KO*^ mice were derived from founders originally obtained from Professor Monty Krieger (Massachusetts Institute of Technology) and backcrossed >10 generations to C57BL/6J mice (50). C57BL/6J wild-type, *ApoE*^*KO/KO*^, and S1PR1-enhanced green fluorescent protein (eGFP) knock-in mice (referred to as *S1pr1*^*eGFP/eGFP*^ in which eGFP was inserted in the coding sequence of the *S1pr1* gene, leading to the expression of a fully functional S1PR1-eGFP fusion protein (51)) were each bred from founders originally purchased from Jackson Laboratories (Bar Harbor, ME). *S1pr1*^*eGFP/eGFP*^*/ApoE*^*KO/KO*^ mice were generated by breeding *S1pr1*^*eGFP/eGFP*^ mice to *ApoE*^*KO/KO*^ mice. The double heterozygous offspring were each intercrossed to generate double-homozygous mice, which were then bred to generate the mice used for experiments.

### Cell preparation culture and treatment

Peritoneal macrophages were elicited by intraperitoneal injection of 1 ml of 10% thioglycolate (Millipore Sigma Canada, Oakville, ON, Canada). At day 4 post-injection, mice were anesthetized with isoflurane and then euthanized by CO_2_ asphyxiation and cervical dislocation. Macrophages were collected by peritoneal lavage using 10 ml of PBS containing 5 mM of EDTA, then centrifuged at 1,200 *g* for 5 min, and resuspended in DMEM supplemented with 10% FBS, 2 mM L-glutamine, 50 μg/ml penicillin, and 50 U/ml streptomycin. Macrophages were counted by hemocytometer and plated in either 8-well chambered slides at 0.7 × 10^5^ cells per 0.8 cm^2^ well for Duolink in situ proximity ligation assay (PLA) or in 6-well culture plate at 2 × 10^6^ cells per 9.5 cm^2^ well for immunoblotting. Cells were then cultured at 37°C in an atmosphere of 5% CO_2_ in air for 2 h allowing macrophages to adhere. Macrophages were then washed at least two times with 1× PBS and incubated for 16 h in DMEM containing 10% FBS, 2 mM L-glutamine, 50 μg/ml penicillin, and 50 U/ml streptomycin.

For Duolink in situ PLA, macrophages were incubated with DMEM supplemented with 3% newborn calf lipoprotein-deficient serum, 2 mM L-glutamine, 50 μg/ml penicillin, and 50 U/ml streptomycin, for 16 h prior to treatment. On the next day, cells were pre-treated for 45 min or 1 h at 37°C with immunological or pharmacological inhibitors followed by HDL addition to macrophages for 30 min. For some experiments, HDL was first pre-treated with recombinant active human S1PL (0.02 μg/1 mg of HDL protein; Millipore Sigma Canada, Oakville, ON, Canada; catalog # SRP0191) or vehicle for 1 h at 37°C before adding it to the cells for 30 min. In other experiments, cells were pre-treated for 45 min with SR-B1 blocking antibody or BLT-1 before adding the HDL and incubation for an additional 30 min. The following compounds were used: anti-SR-B1 blocking antibody, KKB-1, 1.5 μg/ml; originally generated by Karen Kozarsky, SwanBio Therapeutics and provided by Monty Krieger, Massachusetts Institute of Technology) (52); BLT-1 (150 nM, Millipore Sigma Canada, Oakville, ON, Canada; catalog #SML0059); S1P (10 nM, Avanti Polar Lipids, Inc, Birmingham AL; catalog # 860492P); HDL (100 μg/ml; Athens Research And Technology, Athens, GA; catalog #12-16-080412); SEW2871 (1 μM, Cayman Chemicals, Ann Arbor, MI; catalog #10006440), and Ex26 (10 μM, Tocris Bioscience, Bio-Techne Canada, Toronto, ON, Canada; catalog #5833/10). Control cells were treated with corresponding dilutions of solvent/vehicle. Immediately after treatment, macrophages in 8-well chambered slides were washed twice with 1× PBS. The cells were then fixed with 4% paraformaldehyde (PFA) diluted in 1× PBS for 20 min at room temperature (RT), washed three times with 1X PBS, and then Duolink in situ PLA was performed (as described below).

### HDL analysis

HDL that was treated with S1PL or vehicle for 1 h at 37°C, as described above, was fractioned by gel filtration fast-protein liquid chromatography using an AKTA system with a Tricorn Superose 6 HR10/300 column and in line UV absorbance detector (GE Healthcare Life Sciences, Baie D’Urfe, QC, Canada) and 100 μl fractions were collected. Cholesterol levels on each fraction were analyzed by the Infinity Cholesterol enzymatic assay kit (Thermo Fisher Scientific, Ottawa, ON, Canada; catalog #TR13421) to measure total cholesterol. Afterward, the fractions corresponding to the cholesterol peaks were pooled by sets of five fractions starting from fraction 27 to fraction 51. This was done by taking 2.1 μl from each fraction. These five pools of fractions (10 μl of each pool) were subjected to SDS-PAGE immunoblotting with either goat anti-human ApoA1 antiserum (Midland Bioproducts, Nittobo America Inc, Murrieta CA; catalog #71107) or mouse monoclonal anti-ApoM (8F12) antibody (Cell Signaling Technology, Danvers, MA; catalog #5709) and detection was carried out as described below.

### Immunoblotting

Macrophages were washed twice with 1× PBS and then lysed with ice cold RIPA buffer (50 mM Tris-HCl pH-7.4; 150 mM NaCl; 1% Triton X-100; 1% sodium deoxycholate; 0.1% SDS; 1 mM EDTA) supplemented with protease inhibitors containing 1 mM phenylmethylsulfonyl fluoride (Thermo Fisher Scientific, Ottawa, ON, Canada; catalog #36978), 1 μg/ml pepstatin A, 1 mg/ml leupeptin, and 2 μg/ml aprotinin (Millipore Sigma, Canada, Oakville, ON, Canada; catalogs # P5318, L2884, and A1153). Cell lysates were centrifuged for 20 min at 12,000 *g*. Protein concentration was determined from supernatants using the Pierce BCA protein assay kit (Thermo Fisher Scientific, Ottawa, ON, Canada). Afterward, 30 μg protein per lane were mixed with Invitrogen Novex NuPAGE LDS Sample Buffer 4× (Thermo Fisher Scientific, Ottawa, ON, Canada; catalog # NP0007) and incubated at RT for 25 min and immediately subjected to PAGE and immunoblotting. PAGE was carried out using precast NuPAGE 4–12% Bis–Tris gradient gels (Thermo Fisher Scientific, Ottawa, ON, Canada; catalog # NP0321BOX) with 1× MOPS SDS running buffer according to the manufacturer’s instructions. Gels were then electrophoretically transferred to a polyvinylidene fluoride membrane, using a transfer buffer containing 480 mM of Tris, 390 mM of glycine, and 0.375% SDS, at 250 mA for 90 min at 4°C. Membranes were blocked with 5% BSA (New England Biolabs Canada, Whitby, ON, Canada; catalog # 9998S) diluted in TBS containing 0.1% Tween-20 for 1 h at RT. The following antibodies were then used: rabbit anti-SR-B1 (NB400-104; Novus Biologicals), Goat anti-GFP (Abcam Inc, Boston, MA; catalog # ab6673), and HRP-conjugated rabbit anti-β-Actin (Cell Signaling Technology, Danvers, MA; catalog # 5125S). After overnight incubation at 4°C, membranes were washed and incubated with either HRP-donkey anti-rabbit IgG (Jackson Immunoresearch Laboratories, West Grove, PA; catalog #711-035-152), HRP-rabbit anti-goat IgG (Jackson Immunoresearch Laboratories, West Grove, PA; catalog # 305-035-003), or HRP-donkey anti-mouse IgG (Jackson Immunoresearch Laboratories, West Grove, PA; catalog #715-035-150) at 1:5,000 dilution for 1 h at RT. HRP was detected using the Pierce Enhanced Chemiluminescence Western Blotting Substrate (Thermo Fisher Scientific, Ottawa, ON, Canada; catalog # 32106) and a ChemiDoc imaging system (Bio-Rad Laboratories, Hercules, CA).

### Atherosclerosis analysis

We fed *ApoE*^*KO/KO*^ and *S1pr1*^*eGFP/eGFP*^*/ApoE*^*KO/KO*^ mice a high-fat diet (21% butter fat and 0.15% cholesterol, Dyets Inc, Bethlehem PA; catalog #112286), beginning at 15 weeks of age, for eight weeks. Twelve hours before harvest, *S1pr1*^*eGFP/eGFP*^*/ApoE*^*KO/KO*^ mice received intraperitoneal injections of Ex26 (30 mg/kg; n = 4 per group) dissolved in dimethyl sulfoxide (DMSO). Control mice received only DMSO vehicle control (n = 4). Mice were simultaneously fasted for 12 h prior to undergoing isoflurane anesthesia and euthanasia. Hearts were perfused through the left ventricle with 0.9% NaCl containing 10 U of heparin/ml. Hearts were then harvested and frozen in Shandon Cryomatrix embedding medium (Thermo Fisher Scientific, Ottawa, ON, Canada) and stored at −80°C for further analysis. Atherosclerotic plaques from serial cross-sections (10 μm thickness) of the aortic sinuses were stained with oil red O and counterstained with Meyer’s Hematoxylin solution as previously described (5, 6, 30). The cryosections were mounted with CLEAR-MOUNT (BioMeda Corporation, CA; catalog #17985-15). Images were obtained using a Zeiss Axiovert 200 M microscope (Carl Zeiss Canada Ltd Toronto, ON, Canada) at 5× magnification.

### Proximity ligation assay

PLA was performed using Duolink In Situ Detection Reagents Red kit (Millipore Sigma Canada, Oakville ON, Canada; catalog #DUO92008) to examine interactions of endogenous SR-B1 and S1PR1-GFP in primary peritoneal macrophages from *S1pr1*^*eGFP/eGFP*^ knock-in mice and sections of atherosclerotic plaques from *S1pr1*^*eGFP/eGFP*^*/ApoE*^*KO/KO*^ mice. Cultured cells and histological sections were first fixed with 4% PFA as described above. When macrophages were co-stained with Alexa-488 conjugated wheat germ agglutinin (WGA; Thermo Fisher Scientific, Ottawa ON, Canada; catalog # W11261), they were first washed 3× with TBS for 3 min, blocked with 2% BSA in TBS for 20 min, washed 3× with TBS for 3 min, incubated with Alexa 488-WGA diluted 1:500 in 2% BSA in TBS for 15 min, and washed 2× with TBS for 2 min. At this point, WGA-stained cells as well as unstained macrophages and histological sections were permeabilized on ice for 10 min with 0.1% Triton X-100. Samples were then blocked with Duolink® Blocking Solution in a heated humidity chamber for 1 h at 37°C. Afterward, the slides were incubated overnight at 4°C with the following primary antibodies: a goat polyclonal anti-GFP antibody (1:100; Abcam Inc Boston MA; catalog # ab6673) and a rabbit polyclonal anti-SR-B1 antibody (1:100; Bio-Techne Canada, Toronto ON, Canada; catalog # NB400-104), and for some experiments, when cells or histological sections were co-stained for either caveolin-1 or Mac3, respectively, with a mouse monoclonal anti-caveolin 1 (1:500; Bio-Techne Canada, Toronto ON, Canada; catalog # NBP3-23193) or a mouse monoclonal anti-Mac3 antibody (1:200; BD Biosciences; catalog # 553322). Afterward, samples were washed three times with Duolink® wash buffer A (10 mM Tris, pH-7.4, 150 mM NaCl, and 0.05% Tween; Millipore Sigma Canada, Oakville ON, Canada; catalog # DUO82047) under gentle agitation. Anti-rabbit MINUS (Millipore Sigma Canada, Oakville ON, Canada; catalog # DUO92005) and anti-goat PLUS (Millipore Sigma Canada, Oakville ON, Canada; catalog #DUO92003) oligonucleotide-labeled secondary antibodies were added. After 1 h of incubation at 37°C, the slides were washed three times with Duolink® wash buffer A at RT. Slides were then incubated for 30 min at 37°C with the Duolink® ligation mix prepared by diluting 5× Duolink® Ligation buffer (1:5) and ligase (1:40) in water, followed by three washes with Duolink® wash buffer A. Next, the slides were incubated for 100 min at 37°C with the amplification solution prepared by diluting 5× Duolink® Amplification buffer (1:5) and rolling circle polymerase (1:80) in water. For experiments in which cells or tissue sections were co-stained for caveolin-1 or Mac3, respectively, slides were incubated with Alexa488-labeled goat anti-mouse secondary antibody (Thermo Fisher Scientific Canada, Ottawa, ON, Canada; catalog # A21042) for 1 h at room temperature. The slides were then washed two times with Duolink® Wash Buffer B (200 mM Tris, pH-7.5, 100 mM NaCl; Millipore Sigma Canada, Oakville ON, Canada; catalog # DUO82048) and one time with 0.01× Duolink® Wash Buffer B. Finally, the slides were mounted with Duolink® PLA Mounting Medium with DAPI (Millipore Sigma Canada, Oakville ON, Canada; catalog #DUO82040) for 15 min at RT and imaged using Stellaris 5 Confocal Microscope from Leica Microsystems with either a 20× or 63× objective for cells and 63× objective for histological sections of atherosclerotic plaques. For quantification of PLA staining in cultured cells, 3–5 20× fields of view were captured, while for atherosclerotic plaques, 3 63× fields of view were captured for atherosclerotic plaques from each of three sections per mouse. Images were analyzed using Image J software. For analyses of PLA staining, images of the red (PLA) and blue (DAPI) channels were each converted to 8 bit format and thresholds were set using the Image J “Threshold” function to eliminate background noise. Parameters such as size and circularity of particles were set using the “Analyze Particles” function of ImageJ to specify the characteristics of the particles included in the analysis. For quantification of cells stained for PLA and DAPI and imaged at 20× magnification, each particle identified in the PLA channel corresponded to a cell positive for PLA staining and each particle identified in the DAPI channel corresponded to a nucleus. The number of PLA-positive cells across the 3–5 fields of view were divided by the number of DAPI-stained nuclei across the same number of fields of view to obtain a percentage of the cells that were positive for PLA staining. For quantification of PLA staining in atherosclerotic plaques imaged at 63× magnification, particles identified from the PLA channel corresponded to foci of PLA staining in cells. The extent of PLA staining was therefore expressed as the numbers of foci of PLA staining across three fields of view divided by the numbers of DAPI-stained nuclei in the same three fields of view to obtain the number of PLA staining foci per cell.

### Cholesterol efflux

Thioglycolate-elicited peritoneal macrophages were collected from wild-type C57BL/6J mice, cultured in 96-well plates in RPMI containing 10% FBS, 2 mM L-glutamine, 50 μg/ml penicillin, and 50 U/ml streptomycin, for 16 h before changing the medium to phenol red-free RPMI containing 3% newborn calf lipoprotein-deficient serum, 2 mM L-glutamine, 50 μg/ml penicillin, and 50 U/ml streptomycin and culturing for an additional 16 h as described above. Following the manufacturer's instructions, cholesterol efflux was measured using the cell-based fluorescent Cholesterol Efflux Assay kit (Abcam Inc, Boston MA; catalog # ab196985). Cells were preloaded with the fluorescent tracer for 1 h, after which the loading solution was removed, cells were washed, and efflux was initiated by adding the efflux acceptor. Efflux was monitored using either no acceptor, untreated HDL, S1PL-treated HDL, or control-treated HDL (all at 100 μg protein/ml) as cholesterol acceptors. Some cells were also treated with Ex26 (10 μM) or BLT-1 (150 nM) during efflux. In addition, blank wells containing incubation medium without fluorescent cholesterol labeling reagent were included as controls. At the end of 3 h, media was collected, cells were lysed, and fluorescence (excitation 485 nm/emission 523 nm) was determined in each using a SpectraMax 3 fluorescence plate reader. The fluorescence of media from unloaded wells was subtracted from the fluorescence of media from cells loaded with the fluorescent cholesterol tracer to account for the intrinsic background fluorescence of the media. The percentage of cholesterol efflux was calculated as the ratio between the corrected RFU of the media to the sum of the corrected RFU of the media + RFU of the cell lysate × 100%.

### Apoptosis

Thioglycolate-elicited peritoneal macrophages were collected from wild type C57BL/6J mice, cultured in eight well chamber slides in DMEM containing 10% FBS, 2 mM L-glutamine, 50 μg/ml penicillin, and 50 U/ml streptomycin, for 16 h prior to changing the medium to DMEM containing 3% newborn calf lipoprotein-deficient serum, 2 mM L-glutamine, 50 μg/ml penicillin, and 50 U/ml streptomycin, for an additional 16 h as described above. Cells were then cultured for 24 h in the absence or presence of tunicamycin (10 μg/ml), HDL (50 μg protein/ml), BLT-1 (150 nM), or combinations of those as described. Alternatively, cells were incubated in the absence or presence of tunicamycin and either S1PL-treated or control-treated HDL (as described above). After 24 h, cells were fixed in 4% PFA in PBS for 15 min at room temperature, washed with PBS twice, and subjected to Terminal deoxynucleotidyl transferase-mediated dUTP Nick End Labeling (TUNEL) using the ApopTag® Fluorescein In Situ Apoptosis Detection Kit (Millipore Sigma Canada, Oakville, ON, Canada; catalog #S7110) following the manufacturer's instructions. Slides were washed in PBS and counterstained with DAPI (300 nM) for 5 min. The slides were then washed and mounted with microscope slide cover glass by using PermaFluor™ aqueous mounting medium (Thermo Fisher Scientific, Ottawa, ON, Canada; catalog # TA-030-FM). After mounting the slides, they were imaged using a Zeiss Axiovert 200 M inverted fluorescence microscope (Carl Zeiss Canada Ltd. Toronto, ON, Canada) using a 40× objective. Four fields were imaged from each well and the total number of TUNEL-positive nuclei were counted across the four fields and divided by the total number of nuclei as detected by DAPI to determine the % TUNEL-positive cells in each well.

### Statistical analysis

Data was analyzed utilizing GraphPad Prism 6 software (San Diego, CA). Nonparametric Kruskal-Wallis ANOVA test for multiple groups was used. Data were presented as mean ± SEM and were considered statistically significant when *P* <0.05.

## Results

### HDL stimulates the interaction between SR-B1 and S1PR1-eGFP in macrophages

**Fig. 1:**
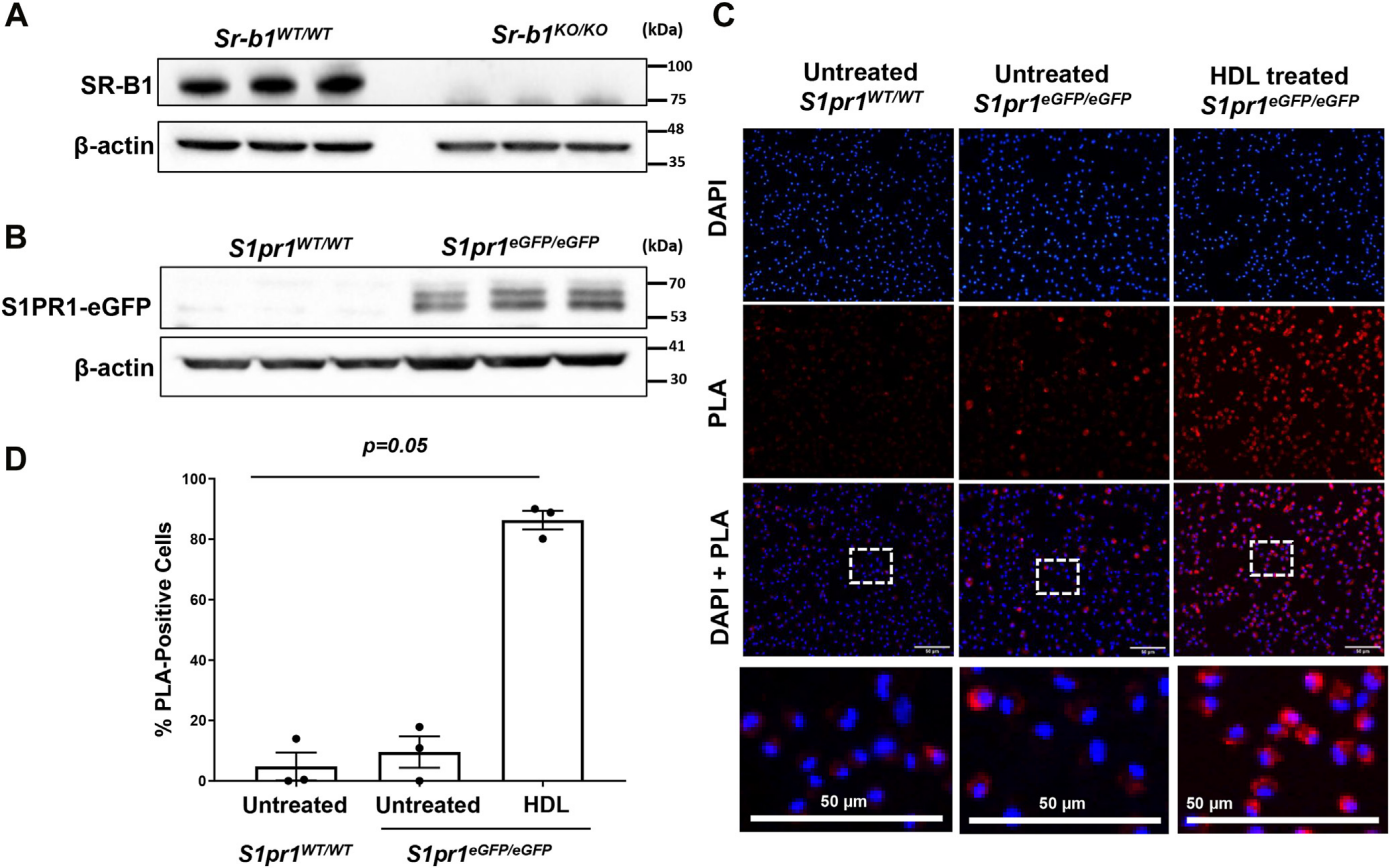
SR-B1 interacts with S1PR1-eGFP in murine macrophages in an HDL-dependent manner. Thioglycolate-elicited peritoneal macrophages were prepared from (A) *Sr-b1*^*WT/WT*^ (C57BL/6J) and *Sr-b1*^*KO/KO*^ mice and (B–D) *S1pr1*^*WT/WT*^ (C57BL/6J) and *S1pr1*^*eGFP/eGFP*^ mice and cultured. A, B: Cells were lysed and protein extracts were prepared and analyzed by immunoblotting for SR-B1, the GFP tag on S1PR1-GFP, and β-actin. C: Macrophages were either untreated or treated in culture with 100 μg (protein)/ml HDL as indicated. Control cells were treated with an equivalent volume of vehicle. After 30 min, cells were fixed, and Duolink proximity ligation assay (PLA) was performed (red fluorescence) as described in the ‘Materials and methods’ section. Nuclei were stained using DAPI (blue). C: Representative images (scale bars represent 50 μm; bottom row shows zoomed-in view of the boxed areas of the DAPI + PLA merged images) and (D) quantification of PLA signal performed by counting the proportion of cells exhibiting PLA signal across three fields of view for each sample well. Data are means ± SEM of n = 3 samples. Each data point represents cells isolated from a different mouse. Data were analyzed using the Kruskal-Wallis test and the *P*-value is indicated. eGFP, enhanced green fluorescent protein; PLA, proximity ligation assay; S1PR, sphingosine-1-phosphate receptor; SR-B1, scavenger receptor class B, type I.

To examine if SR-B1 and S1PR1, expressed at endogenous levels in murine macrophages, physically interact with each other in an HDL dependent-manner, we made use of *S1pr1*^*eGFP/eGFP*^ mice in which *eGFP* was knocked in-frame into the *S1pr1* gene generating an S1PR1-eGFP fusion protein expressed at endogenous S1PR1 levels (51). This allowed us to use eGFP as a C-terminal epitope tag for the S1PR1-eGFP fusion protein. SR-B1 was detected by immunoblotting using an antibody against the C-terminal cytoplasmic tail of SR-B1 in extracts from macrophages from C57BL/6J (*Sr-b1*^*WT/WT*^) but not *Sr-b1*^*KO/KO*^ mice (Fig. 1A). Likewise, using an antibody against the eGFP tag of S1PR1-eGFP, it was detected by immunoblotting in the extracts of primary macrophages from *S1pr1*^*eGFP/eGFP*^ knock-in but not C57BL/6J (*S1pr1*^*WT/WT*^) mice (Fig. 1B). This confirmed the specificity of the antibodies.

Therefore, we used the Duolink in situ PLA assay to study the molecular interaction between SR-B1 and S1PR1-eGFP. This technique generates a red fluorescent signal only when SR-B1 and S1PR1-eGFP are in close (<40 nm) proximity to each other. Peritoneal macrophages isolated from *S1pr1*^*eGFP/eGFP*^ knock-in and WT control mice (lacking the eGFP tag on S1PR1) were treated for 30 min in the presence or absence of HDL and subjected to the Duolink in situ PLA assay. In the absence of HDL, there was little detectable PLA signal in WT macrophages lacking the eGFP tag on S1PR1, whereas a low level of signal was detected in *S1pr1*^*eGFP/eGFP*^ knock-in macrophages. In contrast, *S1pr1*^*eGFP/eGFP*^ knock-in macrophages in the presence of HDL exhibited a robust PLA signal (Fig. 1C, D), indicating that HDL stimulates the physical interaction between SR-B1 and S1PR1 receptors expressed endogenously in mouse primary macrophages in culture.

### HDL-associated S1P promotes the interaction between SR-B1 and S1PR1

**Fig. 2:**
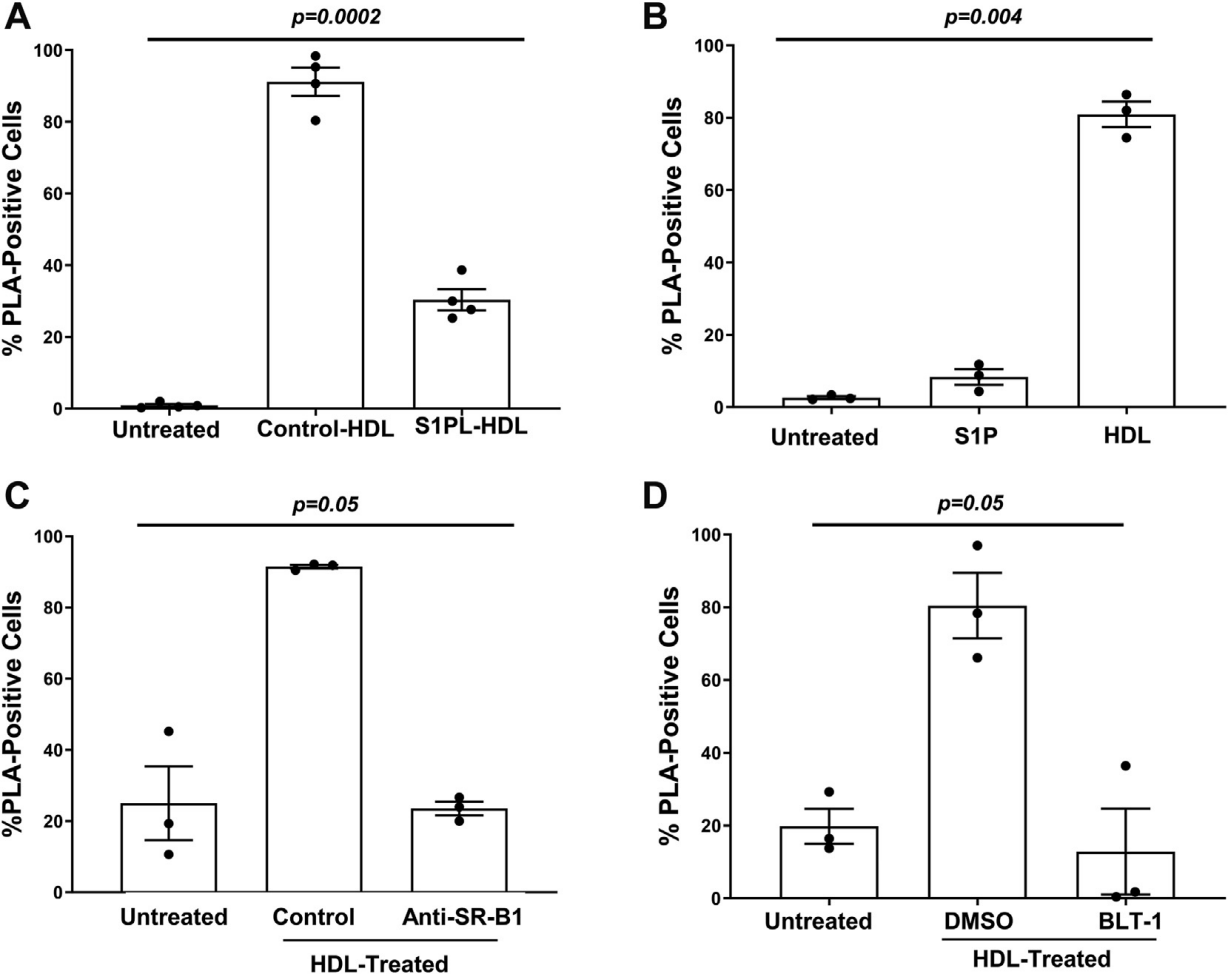
The HDL-stimulated interaction between SR-B1 and S1PR1 is inhibited by S1PL treatment and by inhibition of SR-B1. Thioglycolate-elicited peritoneal macrophages were prepared from *S1pr1*^*eGFP/eGFP*^ mice. A: Cells were incubated for 30 min with HDL that had been pre-treated for 60 min at 37°C with S1PL or with control HDL pre-treated in parallel without S1PL. B: Cells were incubated for 30 min with either S1P (10 nM) or HDL (100 μg protein/ml). C: Cells were pre-treated in culture for 45 min with either an SR-B1 blocking rabbit antiserum or a control, non-immune rabbit antiserum (each at 1.5 μg/ml), as indicated, before the addition of HDL (100 μg protein/ml). D: Cells were pre-treated for 45 min with either BLT-1 (150 nM added in DMSO) or an equivalent amount of DMSO vehicle control. HDL was then added. For each experiment, control cells were also incubated in media in the absence of added HDL (Untreated). After addition of HDL or vehicle, cells were incubated at 37°C for 30 min prior to being washed, fixed, and subjected to the Duolink PLA assay and staining for DAPI. Quantification of PLA signal was performed by counting the proportion of cells exhibiting PLA signal across five fields of view for each sample well. Data are means ± SEM of n = 4 (A) or n = 3 samples (B–D), where each replicate represents cells isolated from a different mouse. Data were analyzed using the Kruskal-Wallis test. *P*-values are indicated. eGFP, enhanced green fluorescent protein; PLA, proximity ligation assay; S1P, sphingosine-1-phosphate; S1PR, sphingosine-1-phosphate receptor; S1PL, S1PL-lyase; SR-B1, scavenger receptor class B, type I.

To test if S1P was required for the interaction between S1PR1 and SR-B1, HDL was pre-treated for 1 h with S1PL prior to incubation with cells. In parallel, control HDL was mock treated in the absence of S1PL prior to adding it to the cells. In a separate experiment, we confirmed that S1PL treatment of HDL did not affect its elution profile on gel-filtration chromatography, as measured by absorbance at 280 nm, for protein, total cholesterol assays of fractions, or immunoblotting for apoA1 (the main structural protein on HDL) or apoM (the carrier of S1P on HDL) (supplemental Fig. 1A–D). The stimulation of the molecular interaction between SR-B1 and S1PR1-eGFP by HDL was significantly reduced when HDL was pre-treated with S1PL compared to control HDL (Fig. 2A). In a separate experiment, we examined if S1P alone could promote the interaction between these two receptors. After incubating the *S1pr1*^*eGFP/eGFP*^ macrophages with S1P for 30 min, a slight increase in the average PLA signal was apparent, but this did not reach statistical significance. In parallel, *S1pr1*^*eGFP/eGFP*^ macrophages incubated for 30 min with HDL showed a robust increase in the PLA signal (Fig. 2B). These results demonstrate that S1P is necessary for HDL to fully stimulate the interaction between SR-B1 and S1PR1-eGFP in macrophages and that S1P alone (in the absence of HDL) is not sufficient to stimulate the interaction.

### Inhibition of SR-B1 attenuates the HDL-stimulated interaction between SR-B1 and S1PR1

To test the involvement of the extracellular domain of SR-B1 in the HDL-stimulated interaction between SR-B1 and S1PR1-eGFP, peritoneal macrophages from *S1pr1*^*eGFP/eGFP*^ mice were pre-treated for 45 min with an antibody (rabbit anti-mSR-B1 KKB-1 antibody), against the extracellular domain of SR-B1 that was previously reported to block HDL binding (52). As a control, cells were treated in parallel with a non-immune anti-rabbit IgG antibody. HDL (100 μg protein/ml) was added and SR-B1/S1PR1-eGFP complex formation was measured using the Duolink PLA assay. Pre-incubation with the anti-SR-B1 blocking antibody significantly reduced the PLA signal compared to cells pre-incubated with the control IgG antibody (Fig. 2C), demonstrating the involvement of SR-B1’s extracellular domain in the HDL-stimulated interaction with S1PR1. It is not clear if this reflected immunological blockade of SR-B1’s ability to bind HDL (thereby preventing HDL-stimulation) or stearic hindrance of the interaction between SR-B1 and S1PR1. We therefore explored the effect of BLT-1, a small molecule inhibitor of SR-B1’s ability to transfer lipids between the bound HDL and cells. BLT-1 has been reported to covalently modify a cysteine side chain protruding into the hydrophobic channel of SR-B1 resulting in the inhibition of SR-B1-mediated lipid transport but not HDL binding (22, 25, 53). Macrophages from *S1pr1*^*eGFP/eGFP*^ mice were pre-treated with 150 nM BLT-1, before the addition of HDL (100 μg protein/ml). Control cells were incubated with DMSO as a vehicle. BLT-1 treatment of cells prevented HDL from stimulating the association of SR-B1 with S1PR1-eGFP (Fig. 2D), suggesting that SR-B1-mediated lipid transfer activity is necessary to trigger the interaction between these two receptors in macrophages.

### SEW2871, an S1PR1-selective agonist, promotes and Ex26, an S1PR1-selective antagonist, inhibits the interaction between SR-B1 and S1PR1

**Fig. 3:**
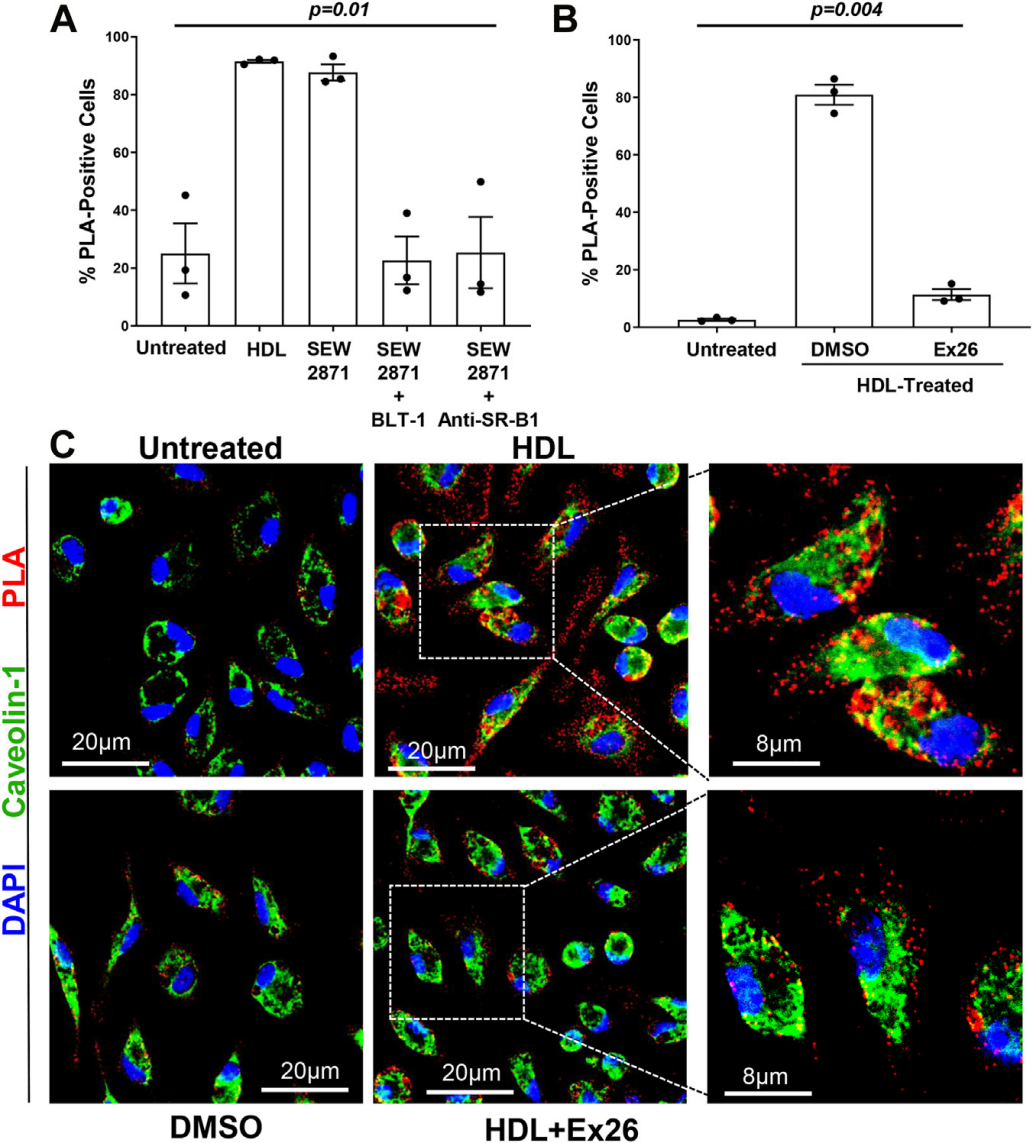
The S1PR1 selective agonist, SEW2871, stimulates and the selective antagonist Ex26 inhibits the interaction between SR-B1 and S1PR1. Thioglycolate-elicited peritoneal macrophages from *S1pr1*^*eGFP/eGFP*^ mice were (A) pre-treated with either the anti-SR-B1 antiserum (1.5 μg/ml) or the SR-B1 inhibitor BLT-1 (150 nM) for 45 min or with control non-immune rabbit serum and DMSO vehicle (where not indicated). After the pre-treatment period, HDL (100 μg protein/ml) or the S1PR1 selective agonist, SEW2871 (1 μM) or no stimulus (untreated) was added for an additional 30 min before cells were fixed. B: Cells were treated with either Ex26 (10 μM, added as a 1,000× stock in DMSO) or DMSO vehicle control for 60 min, at which point HDL (100 μg protein/ml) or vehicle was added and cells were incubated for a further 30 min. After the 30 min incubation with HDL, cells were washed, fixed, and subjected to Duolink PLA and DAPI staining, imaging, and analysis as in the legend to Figs. 1 and 2. Each symbol represents cells isolated from a different mouse. Data were analyzed using Kruskal-Wallis test; *P*-values are indicated above each graph. C: Thioglycolate-elicited peritoneal macrophages from *S1pr1*^*eGFP/eGFP*^ mice were incubated for 60 min with 10 μM Ex26 or DMSO vehicle control followed by treatment without or with HDL (100 μg protein/ml) for 30 min as indicated. Cells were then washed and fixed, and PLA staining (red) was carried out followed by staining for caveolin-1 using a mouse anti-caveolin-1 antibody and Alexa488-conjugated anti-mouse secondary antibody (green) and DAPI staining (blue). Panels on the right show zoomed-in views of the boxed areas of the merged images of HDL-treated control and Ex26-treated cells. eGFP, enhanced green fluorescent protein; PLA, proximity ligation assay; S1PR, sphingosine-1-phosphate receptor; SR-B1, scavenger receptor class B, type I.

We examined the effect of treating primary mouse macrophages with SEW2871, a selective agonist of S1PR1, on the physical interaction between SR-B1 and S1PR1. Treatment of macrophages with 1 μM of SEW2871 for 30 min increased the PLA signal between SR-B1 and S1PR1-eGFP to a similar extent as that triggered by HDL (Fig. 3A). To determine if inhibition of SR-B1’s lipid transfer activity affected SEW2871-mediated SR-B1/S1PR1-eGFP interaction, macrophages were pre-treated with or without BLT-1 or with the SR-B1-blocking antibody for 45 min. Cells were then incubated with SEW2871 for 30 min. Interestingly, the PLA signal induced by SEW2871 was significantly reduced by treatment with either BLT-1 or the anti-SR-B1-blocking antibody (Fig. 3A). These findings suggest that SR-B1 activity is required for the SEW2871-stimulated interaction between SR-B1 and S1PR1-eGFP, even in the absence of HDL.

To examine the effect of inhibiting S1PR1 activity on the HDL-stimulated interaction between SR-B1 and S1PR1, we used a selective antagonist for S1PR1 called Ex26 (54). Primary mouse peritoneal macrophages isolated from *S1pr1*^*eGFP/eGFP*^ mice were first pre-treated for 1 h with 10 μM Ex26 or DMSO as a control, followed by incubation with HDL (100 μg protein/ml) for 30 min. Treatment of cells with Ex26 significantly reduced (86%) the HDL-stimulated interaction between SR-B1 and S1PR1-eGFP. This result confirms that the activity of S1PR1 is critical for its molecular interaction with SR-B1 in cultured macrophages (Fig. 3B). To visualize the distribution of HDL-stimulated SR-B1/S1PR1 complexes within macrophages and the effects of Ex26-mediated antagonism of S1PR1, macrophages from *S1pr1*^*eGFP/eGFP*^ mice were either untreated or treated with DMSO solvent control or HDL in the absence or presence of Ex26, fixed, permeabilized, and subjected to the Duolink PLA staining along with staining for caveolin-1 and imaged by confocal microscopy (Fig. 3C). HDL treatment dramatically increased punctate PLA signal throughout the cells in regions that did not coincide with staining for caveolin-1. Ex26 treatment reduced the numbers without noticeably affecting the distribution of PLA-positive punctae. Separate samples were stained with green fluorescently labeled WGA (to label the cell surface) prior to permeabilization and PLA staining, demonstrating similar effects of HDL Ex26 (supplemental Fig. S2).

### Effects of inhibition of SR-B1 or S1PR1 on HDL-mediated cholesterol efflux and protection of macrophages against apoptosis

**Fig. 4:**
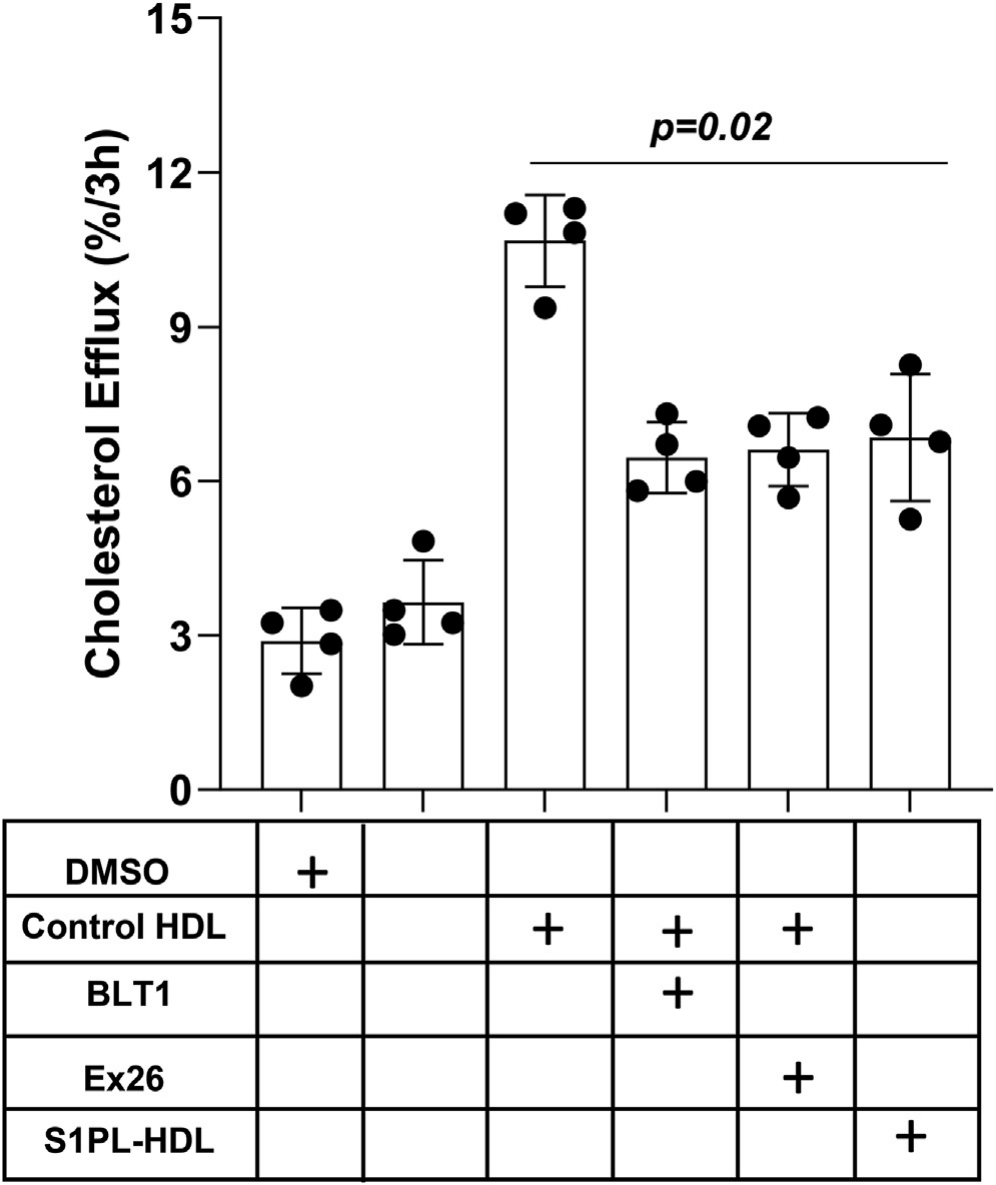
The SR-B1 inhibitor, BLT-1, the S1PR1 antagonist Ex26, and S1PL treatment of HDL all reduce HDL-mediated cholesterol efflux from macrophages. Thioglycolate-elicited peritoneal macrophages were collected from wild type C57BL/6J mice, cultured in DMEM containing 10% FBS, 2 mM L-glutamine, 50 μg/ml penicillin, and 50 U/ml streptomycin, for 16 h prior to changing the medium to phenol red-free DMEM containing 3% newborn calf lipoprotein-deficient serum, 2 mM L-glutamine, 50 μg/ml penicillin, and 50 U/ml streptomycin, for an additional 16 h as described above. Cholesterol efflux was measured using the cell-based fluorescent Cholesterol Efflux Assay kit. After loading cells with the fluorescent tracer for 1 h, cells were washed and efflux was initiated by addition of the efflux acceptor medium. Efflux was monitored using either no acceptor or control- or S1PL-treated HDL (each at 100 μg protein/ml) as cholesterol acceptors. Some cells were also treated with Ex26 (10 μM) or BLT-1 (150 nM) during efflux. Cholesterol efflux was measured as the amount of cholesterol tracer appearing in the medium at the end of 3 h, as a % of the total cholesterol tracer (cells + medium). Each symbol represents cells isolated from a different mouse (n = 4); bars represent means and error bars represent standard errors. Data was analyzed by the Kruskal-Wallis test; *P* value is indicated. S1PL, S1PL-lyase; S1PR, sphingosine-1-phosphate receptor; SR-B1, scavenger receptor class B, type I.

We next wanted to test if these treatments that interfered with HDL-stimulated SR-B1 and S1PR1 complex formation impacted HDL-mediated cholesterol efflux from macrophages. HDL-mediated cholesterol efflux was measured using a cholesterol efflux assay kit in which cells were pre-loaded with a fluorescently labeled analog of cholesterol, and efflux was initiated by the addition of HDL or S1PL-treated HDL for 3 h. Baseline efflux in the absence of added HDL as a cholesterol acceptor or in the presence of DMSO as a solvent control was also measured. This demonstrated that addition of control HDL triggered a substantial 3-fold increase in the efflux of cholesterol after 3 h as compared to basal efflux without a cholesterol acceptor (Fig. 4). In contrast, S1PL treatment of HDL prior to its addition to cells reduced cholesterol efflux by approximately 40% compared to untreated HDL. Treatment of cells with either the S1PR1 antagonist, Ex26, or BLT-1, the inhibitor of SR-B1-mediated lipid transport, resulted in similar levels of inhibition of cholesterol efflux to HDL. This suggests that S1PL treatment and antagonism of S1PR1 are each able to reduce HDL-mediated cholesterol efflux to the same extent as inhibition of SR-B1 but do not impact SR-B1-independent HDL-mediated cholesterol efflux pathways.

**Fig. 5:**
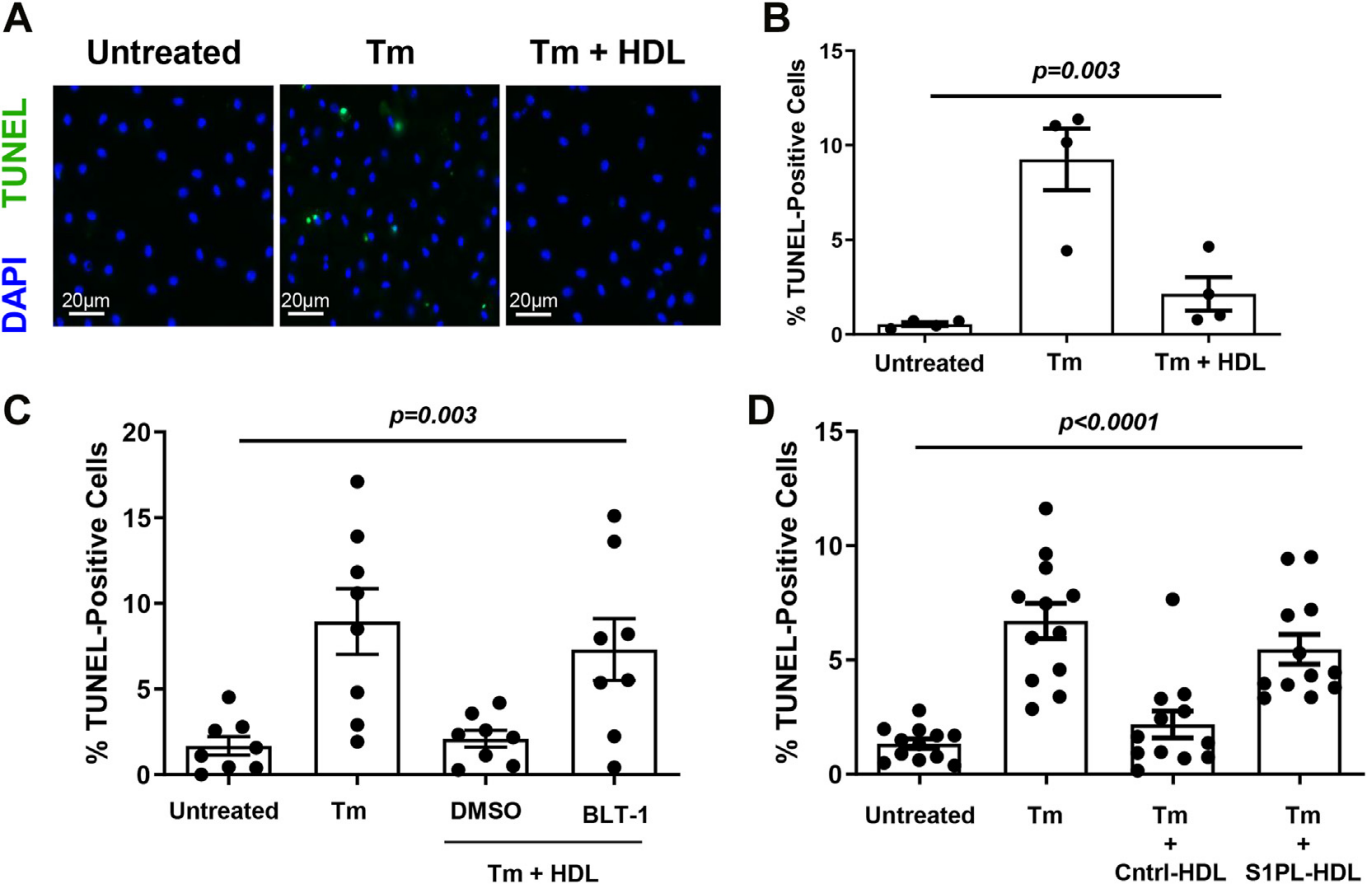
HDL-mediated protection of macrophages from tunicamycin-induced apoptosis is inhibited by the SR-B1 inhibitor BLT-1 and by S1PL-treatment of HDL. Thioglycolate-elicited peritoneal macrophages from wild type C57BL/6J mice were cultured in 8-well chamber slides in DMEM containing 3% newborn calf lipoprotein-deficient serum for 16 h prior to the addition of (A, B) tunicamycin (10 μg/ml) without or with HDL (50 μg protein/ml), (C) tunicamycin (10 μg/ml), HDL (50 μg protein/ml), and BLT-1 (150 nM) or combinations of those as indicated; or (D) tunicamycin (10 μg/ml) and either S1PL-treated or control-treated HDL (50 μg protein/ml) as indicated. After 24 h, cells were fixed and apoptosis was detected by TUNEL staining for fragmented DNA, followed by counter-staining with DAPI for nuclear DNA. A: Representative images of TUNEL (green fluorescence) and DAPI (blue fluorescence) stained nuclei. B–D: Quantification of the degree of apoptosis (% TUNEL-positive nuclei). Each data point in (B) represents cells isolated from a different mouse (n = 4). Data points in (C) and (D) represent independent wells of cells isolated from 2 to 4 mice, plated in triplicate. Data were analyzed by the Kruskal-Wallis test; *P* values are indicated above each graph. S1PL, S1PL-lyase; TUNEL, Terminal deoxynucleotidyl transferase-mediated dUTP Nick End Labeling; SR-B1, scavenger receptor class B, type I.

We have previously demonstrated that HDL can protect macrophages from apoptosis induced by different agents including tunicamycin and thapsigargin, both of which induce ER stress and UV irradiation and that this can be inhibited by knockout of S1PR1 expression (5, 30, 34). Consistent with our previously reported findings, tunicamycin treatment of macrophages cultured in serum depleted of lipoproteins triggered increased apoptosis, measured by TUNEL staining for fragmented DNA (Fig. 5A, B). Co-treatment with HDL prevented the tunicamycin-stimulated apoptosis. However, the SR-B1 inhibitor, BLT-1, largely prevented the HDL-mediated protection of macrophages against tunicamycin-induced apoptosis demonstrating that this property of HDL was dependent on SR-B1 (Fig. 5A, B). S1PL pretreatment of HDL similarly inhibited HDL-mediated protection against tunicamycin-induced macrophage apoptosis (Fig. 5C). These findings demonstrated that treatments which prevented HDL-stimulated SR-B1 and S1PR1 complex formation in macrophages also inhibited SR-B1-dependent, HDL-mediated cholesterol efflux and protection of macrophages against tunicamycin-induced apoptosis.

### SR-B1 and S1PR1 form complexes in macrophages within atherosclerotic plaques

**Fig. 6:**
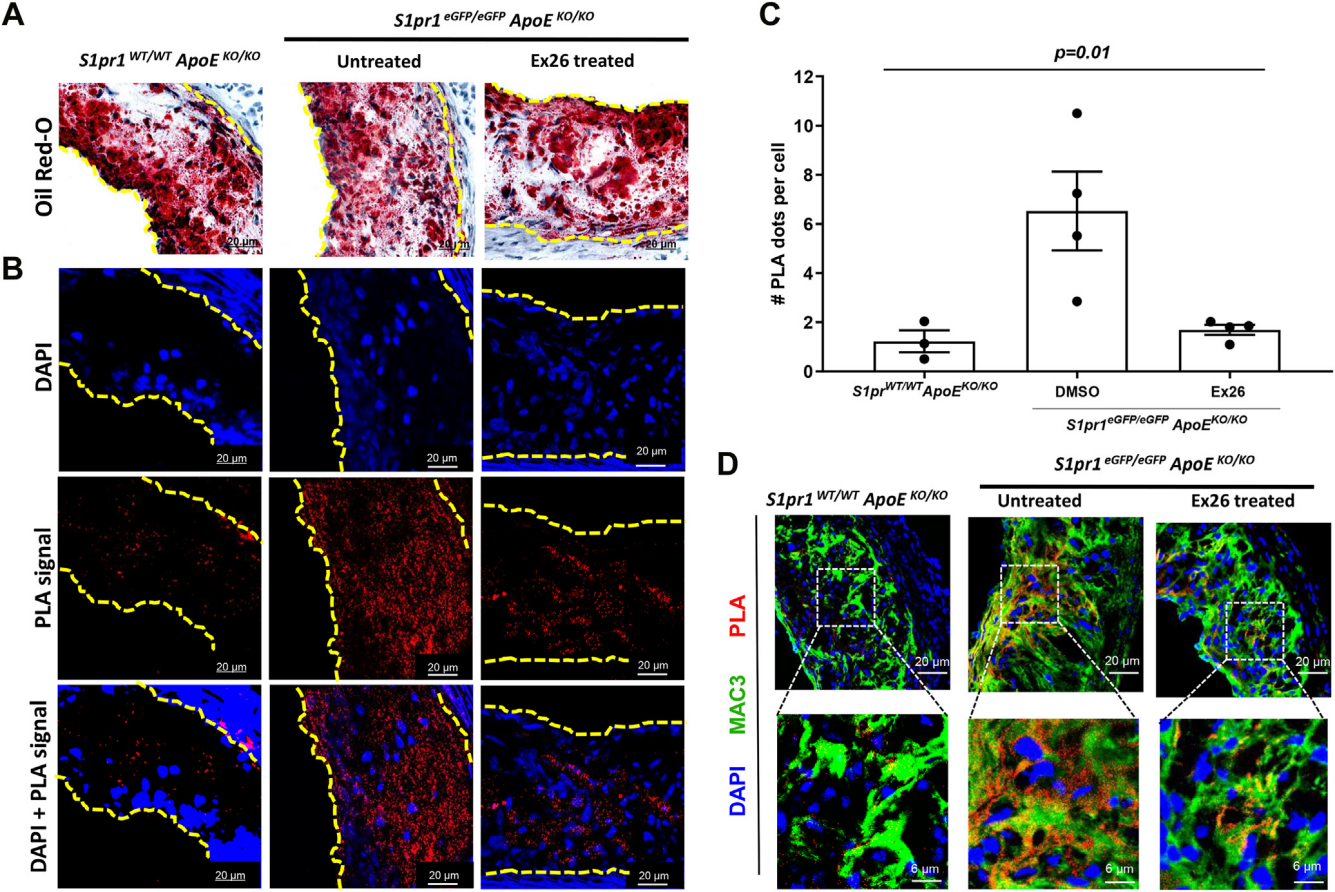
SR-B1 and S1PR1 interact in atherosclerotic plaques of high-fat diet-fed *ApoE*^*KO/KO*^ mice. Control *ApoE*^*KO/KO*^ mice in which S1PR1 was not GFP tagged (*S1pr1*^*WT/WT*^/*ApoE*^*KO/KO*^ mice), and *S1pr1*^*eGFP/eGFP*^*ApoE*^*KO/KO*^ mice were fed a high fat, high cholesterol diet for 8 weeks, beginning at 15 weeks of age. *S1pr1*^*eGFP/eGFP*^/*ApoE*^*KO/KO*^ mice were then treated with Ex26 (30 mg/kg in DMSO) or DMSO vehicle and mice were euthanized, and tissues were harvested 12 h later. Adjacent cross-sections in the aortic sinus were collected and stained with oil red O (lipid) and hematoxylin (nuclei) to detect lipid-rich atherosclerotic plaques or were fixed, permeabilized, and subjected to Duolink PLA staining using antibodies against SR-B1 and GFP as described in the ‘Materials and methods’ section. PLA-stained sections were counterstained with DAPI for nuclear DNA. A: Representative images of oil red O and hematoxylin-stained atherosclerotic plaques in the aortic sinuses. B: Images of adjacent PLA-stained sections of plaques showing DAPI, PLA signal, and merged DAPI and PLA signals. Scale bars (A, B) represent 20 μm. C: Quantification of PLA signal in the atherosclerotic plaques of *S1pr1*^*WT/WT*^*/ApoE*^*KO/KO*^ and DMSO vehicle or Ex26-treated *S1pr1*^*eGFP/eGFP*^*/ApoE*^*KO/KO*^ mice was performed by counting the average of PLA signal punctae within atherosclerotic plaques across three fields of view per plaque of three sections for each mouse and dividing by the number of DAPI-stained nuclei. Each data point represents data from a different mouse. Data were analyzed using the Kruskal-Wallis test; *P* value is indicated above the graph. D: Confocal images of PLA (red) and anti-Mac3 immunofluorescence (green) and DAPI (blue) co-stained images of atherosclerotic plaques from control *S1pr1*^*WT/WT*^*/ApoE*^*KO/KO*^ and DMSO vehicle or Ex26-treated *S1pr1*^*eGFP/eGFP*^*/ApoE*^*KO/KO*^ mice. Images in the bottom row correspond to zoomed-in views of the boxed areas. eGFP, enhanced green fluorescent protein; PLA, proximity ligation assay; S1PR, sphingosine-1-phosphate receptor; SR-B1, scavenger receptor class B, type I.

To examine whether SR-B1 and S1PR1 form complexes in cells in vivo, spleens and adrenal glands were collected from *S1pr1*^*eGFP/eGFP*^ and control *S1pr1*^*WT/WT*^ mice, and PLA staining for SR-B1 and S1PR1-eGFP interactions was carried out on cryosections of these tissues. PLA signal was detected in the sections of spleens (a tissue rich in immune cells, including macrophages) and in adrenal glands (a tissue known to express high levels of both SR-B1 and S1PR1) from *S1pr1e*^*GFP/eGFP*^ but not *S1pr1*^*WT/WT*^ mice, expressing normal untagged S1PR1 (supplemental Fig. S3). To test if SR-B1 and S1PR1 form complexes in cells within atherosclerotic plaques, we generated *S1pr1*^*eGFP/eGFP*^*/ApoE*^*KO/KO*^ mice and fed them a high fat, high cholesterol, atherogenic diet for 8 weeks to promote atherosclerosis development in their aortic sinuses. As a negative control, we included atherogenic diet-fed *ApoE*^*KO/KO*^ mice (referred to as *S1pr1*^*WT/WT*^*/ApoE*^*KO/KO*^ to emphasize that the S1PR1 protein did not contain the eGFP tag). We also treated a subset of the atherogenic diet-fed *S1pr1*^*eGFP/eGFP*^*/ApoE*^*KO/KO*^ mice with the Ex26 to antagonize S1PR1 12 h before harvest (control mice were treated in parallel with DMSO vehicle). Histological sections of atherosclerotic plaques in the aortic sinus from these mice were stained with oil red O (for neutral lipids) and hematoxylin for nuclei, to visualize atherosclerotic plaques (Fig. 6A). Adjacent cryo-sections were subjected to Duolink PLA staining using primary antibodies against the C-terminal cytoplasmic region of SR-B1 and the cytoplasmic GFP tag of S1PR1-eGFP (Fig. 6B). We detected abundant PLA signal in atherosclerotic plaques from *S1pr1*^*eGFP/eGFP*^*/ApoE*^*KO/KO*^ mice as compared to those from *S1pr1*^*WT/WT*^*/ApoE*^*KO/KO*^ mice (Fig. 6B, C). Furthermore, the treatment of *S1pr1*^*eGFP/eGFP*^*/ApoE*^*KO/KO*^ mice with Ex26 significantly reduced PLA signal to background levels seen in the plaques from *S1pr1*^*WT/WT*^*/ApoE*^*KO/KO*^ mice (Fig. 6B, C). Co-staining of sections through atherosclerotic plaques for the macrophage marker Mac3 together with PLA staining for SR-B1 and S1PR1-GFP complexes, together with confocal imaging at high magnification (Fig. 6D), revealed that the PLA signal was largely associated with Mac3-expressing cells within atherosclerotic plaques. These results suggest that SR-B1 and S1PR1-GFP physically interacted in macrophages within the atherosclerotic plaque of experimental mice in a manner that requires activity of S1PR1, consistent with the interactions between SR-B1 and S1PR1 in cultured macrophages.

## Discussion

It has previously been reported that SR-B1 and S1PR1 form ligand-dependent complexes when they are overexpressed in transfected HEK293 cells and that HDL mediated calcium signaling in a manner dependent on both of these receptors (49). However, whether SR-B1/S1PR1 complex formation also occurs at much lower levels of endogenous expression of the two receptors in primary cells in culture or in vivo was not tested. We have previously reported that SR-B1 and S1PR1 are both required for HDL signaling in primary murine macrophages leading to Akt phosphorylation, and others have shown that S1PR1 is required for HDL-mediated activation of signal transducer and activator of transcription 3 in macrophages and that these signaling pathways lead to chemotaxis and/or protection against cell death (5, 6, 28, 30, 35). Therefore, we set out to test if SR-B1 and S1PR1 formed complexes in primary murine macrophages in culture and in atherosclerotic plaques, largely comprised of macrophage-like foam cells. To do this, we made use of the Duolink PLA. This uses antibodies tagged with oligonucleotides: When the antibodies target two members of a protein complex and bring the oligonucleotide tags within 40 nm of each other, they can be ligated and amplified, incorporating fluorescent probes which are visualized by fluorescence microscopy. In order to do this, we screened a number of commercially available antibodies reported to target S1PR1, making use of macrophages from mice with a myeloid-specific *S1pr1* gene knockout that we have previously generated (30). Unfortunately, all the anti-S1PR1 antibodies that we tested exhibited substantial immunofluorescence signal in the macrophages lacking *S1pr1* gene expression (not shown). To overcome the difficulty of finding an antibody suitably specific for S1PR1, we made use of a line of mice in which the complementary DNA for eGFP was knocked in-frame into the *S1pr1* gene such that the modified gene encoded a fusion protein in which the full-length S1PR1 protein contained eGFP fused to its cytoplasmic carboxy-terminus (51). Importantly, these mice express normal endogenous levels of the S1PR1-eGFP fusion protein, which exhibits normal function (51). Using a commercially available antibody against eGFP and an antibody against the cytoplasmic carboxy-terminus of SR-B1, in conjunction with the Duolink PLA, we report that, at endogenous expression levels in primary macrophages in culture and in cells in atherosclerotic plaques, SR-B1 and S1PR1 form complexes in response to HDL or to an S1PR1 agonist, SEW2871. Furthermore, the stimulation of formation of these complexes is blocked by an antibody that binds to the extracellular domain of SR-B1, an inhibitor (BLT-1) which blocks SR-B1’s lipid transport activity, and an antagonist of S1PR1 signaling (Ex26).

The majority of S1P in plasma is carried by HDL (55), and HDL-bound S1P has been reported to act as a biased ligand for S1PR1 in endothelial cells (32). Furthermore, HDL signaling in endothelial cells requires SR-B1 and S1PR1 (32, 37, 56, 57). We reported that HDL stimulation of Akt phosphorylation, macrophage migration, and protection of macrophages against apoptosis are lost when S1PR1 is knocked out or functionally antagonized, suggesting that HDL stimulation of these responses by macrophages are dependent on S1PR1 signaling (28, 30). Likewise, we found that HDL stimulation of SR-B1/S1PR1-GFP complex formation in macrophages in culture is prevented by an antagonist of S1PR1, or by S1PL, an enzyme that irreversibly converts S1P into ethanolamine and a fatty aldehyde, neither of which are able to elicit S1PR1 signaling. This indicates that HDL stimulation of SR-B1 and S1PR1 complex formation is dependent on S1P. In contrast, S1P added in the absence of a lipoprotein carrier did not stimulate SR-B1/S1PR1 complex formation. These results in macrophages are consistent with what was previously reported in HEK293 cells overexpressing both recombinant S1PR1 and SR-B1, where it was shown that S1P is a necessary component of HDL, but this bioactive sphingolipid is not sufficient for promoting the SR-B1/S1PR1 interaction on its own (49). These findings are consistent with the idea proposed by others that HDL-associated S1P acts as a biased ligand for S1PR1, eliciting responses distinct from those elicited by S1P that is not delivered by HDL (32, 58). Unlike S1P, however, the synthetic S1PR1-specific agonist, SEW2871, was able to stimulate SR-B1/S1PR1 complex formation in macrophages in the absence of lipoprotein carriers. However, SEW2871-mediated SR-B1/S1PR1 complex formation was also inhibited by BLT-1. This suggests that SR-B1 may play a role in the delivery of SEW2871 to S1PR1, thereby facilitating its activation.

When HDL pre-treated with S1PL was used or when cells were incubated with the S1PR1 antagonist, Ex26, HDL-mediated cholesterol efflux was reduced to similar levels as that observed when cells were treated with the SR-B1 transport inhibitor, BLT-1. This suggests that S1P contributes to HDL-mediated cholesterol efflux from macrophages, likely as a result of S1PR1 signaling and through SR-B1-mediated cholesterol transport (since the S1PR1 antagonist and the SR-B1 transport inhibitor both reduced HDL-mediated efflux to the same extent as S1PL treatment). S1P has previously been implicated in the regulation of ABCA1-mediated cholesterol efflux through the S1PR3 receptor (59, 60). Our findings demonstrate that S1P similarly regulates HDL-mediated cholesterol efflux through SR-B1 and implicate SR-B1/S1PR1 complex formation in this process. Similarly, HDL-mediated protection against tunicamycin-induced apoptosis was reduced either when HDL was pretreated with S1PL or when cells were incubated with the SR-B1 inhibitor. This suggests that treatments which prevented HDL-mediated SR-B1 and S1PR1 complex formation also impacted HDL-dependent protection against apoptosis, suggesting a role for SR-B1 and S1PR1 complex formation in this process as well.

In addition to demonstrating that SR-B1 and S1PR1 form complexes in primary macrophages in culture, we demonstrated that SR-B1 and S1PR1 form complexes in cells within spleens and in adrenal glands (known to highly express SR-B1) of *S1pr1*^*eGFP/eGFP*^ mice as well as in macrophages within atherosclerotic plaques in *S1pr1*^*eGFP/eGFP*^*/ApoE*^*KO/KO*^ mice fed a high fat diet. Macrophages and macrophage-like foam cells make up the majority of cells within atherosclerotic plaques (2). Furthermore, we demonstrated that treatment of high fat diet-fed *S1pr1*^*eGFP/eGFP*^*/ApoE*^*KO/KO*^ mice with Ex26 disrupted SR-B1/S1PR1 complexes in atherosclerotic plaque macrophages. Ex26 is a specific antagonist of S1PR1 which also reportedly inhibits its internalization and degradation (61). These findings suggest that the activity of S1PR1 is required for its interaction with SR-B1 in macrophages both in culture and within murine atherosclerotic plaques. These findings, along with our demonstration that the disruption of SR-B1/S1PR1 complex formation is associated with reductions in SR-B1-mediated HDL functions including cholesterol efflux and protection against apoptosis, suggest that SR-B1/S1PR1 complex formation in macrophages may play an important role in these atheroprotective pathways in vivo. Our previous demonstration that S1PR1 knockout in myeloid cells, which include macrophages, accelerates diet-induced atherosclerotic plaque development in LDLR-deficient mice is consistent with this (30).

The mechanisms and stoichiometry of SR-B1 and S1PR1 complex formation are unclear and require further investigation. Both SR-B1 and S1PR1 have been reported previously to be at least partially localized to cholesterol-rich lipid rafts or plasma membrane domains enriched in caveolin-1 (20, 62, 63). However, the SR-B1/S1PR1 complexes identified by the PLA signal do not appear to colocalize with caveolin-1 in cultured macrophages, suggesting that they are not located in cholesterol-rich caveolae. SR-B1 and S1PR1 complexes are not confined to macrophages since we have also detected them in the sections of adrenal glands. Adrenocortical cells in the adrenal gland are known to express very high levels of SR-B1 protein and also express S1PR1 (20, 64). The role that SR-B1/S1PR1 complexes play in adrenocortical cell functions such as HDL-cholesterol uptake or steroid hormone biosynthesis remains to be explored.

In conclusion, our study demonstrates that HDL can stimulate the molecular interaction between S1PR1 and SR-B1 proteins expressed at endogenous levels in murine macrophages in culture and within atherosclerotic plaques. Both SR-B1 and S1PR1 have been reported to be required for HDL signaling in macrophages and other cell types. The observation that these receptors form complexes in macrophages in culture and within experimental atherosclerotic plaques provides insights into the mechanisms by which they are both involved in HDL signaling in macrophages and suggests that this may impact atherosclerotic plaque development. We have previously demonstrated that HDL-mediated protection of macrophages against cell death involves S1PR1 (30) and SR-B1 (6), and we and others have reported that KO of either SR-B1 in bone marrow-derived cells (48, 65) or S1PR1 in myeloid cells (30), both of which include macrophages, results in increased cell death in atherosclerotic plaques and increased atherosclerosis development. Similarly, S1PR1 inactivation in endothelial cells also increased atherosclerosis (32). Furthermore, some studies have reported that treatment of mice with S1PR1 agonists reduced atherosclerosis while other studies reported no effects on atherosclerotic plaque size (61, 66, 67, 68). Further research is needed to understand the importance of S1PR1 signaling and the role of SR-B1/S1PR1 complex formation for HDL signaling in macrophages as well as other cell types (endothelial and smooth muscle cells) during atherosclerosis development and whether this represents an opportunity for therapeutic intervention.

## Data availability

The data described in the manuscript are available in the Supplementary materials or from B. Trigatti, McMaster University (trigatt@mcmaster.ca) upon request.

## Supplemental data

This article contains supplemental data.

## Conflict of interest

The authors declare that they have no conflicts of interest with the contents of this article.
