# Supplementary material for: Ligand-dependent interactions between SR-B1 and S1PR1 in macrophages and atherosclerotic plaques
Source: J Lipid Res. 2024 Apr 5;65(5):100541. doi: 10.1016/j.jlr.2024.100541 (PMC11087725; doi:10.1016/j.jlr.2024.100541)
Supplement: Supplemental Figures S1–S3 [file mmc1.pdf]

Ligand-dependent interactions between SR-B1 and S1PR1 in macrophages and atherosclerotic plaques.

Christine Bassila, George E G Kluck, Narmadaa Thyagarajan, Kevin M Chathely, Leticia Gonzalez, Bernardo L Trigatti

Supplementary Figures

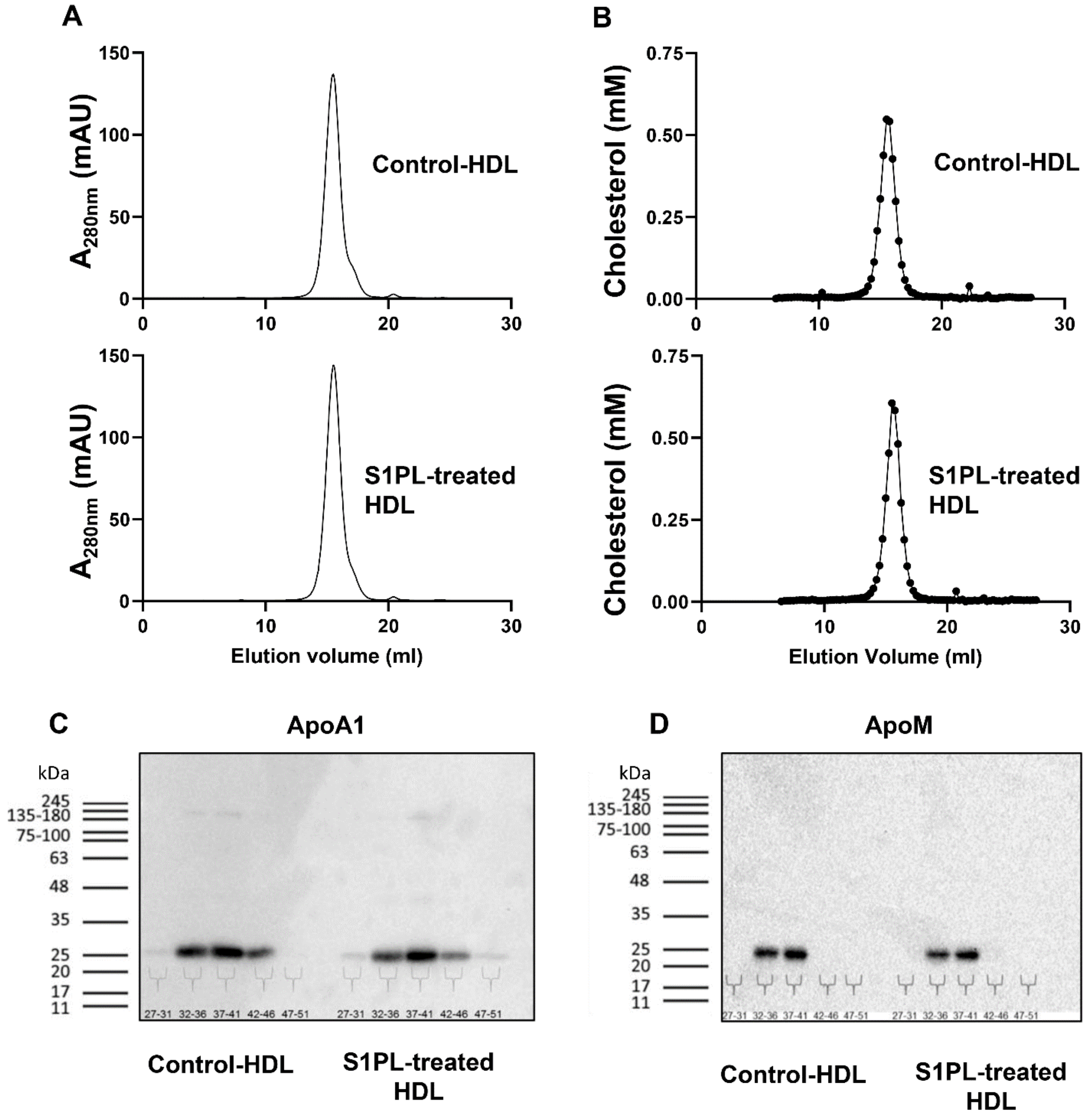

Supplementary Figure 1: Characterization of S1PL-treated HDL by size exclusion chromatography. HDL was incubated for 1 hour at 37°C either without (control-HDL) or with 0.02µg of S1PL per mg of HDL protein (S1PL-Treated HDL). Afterwards, 250 µg (protein mass) of S1PL-treated or control HDL was subjected to size exclusion chromatography on a Tricorn Superose 6 HR 10/300 column using an Akta FPLC system. **A.** UV absorbance profile of eluted protein. Top: Control-HDL. Bottom: S1PL-treated HDL. **B.** Cholesterol content of each fraction. Top: Control-HDL. Bottom: S1PL-treated HDL. Immunoblotting for **C.** ApoA1 and **D.** ApoM in pooled fractions. Fractions from # from 27 to 51 were pooled in sets of 5 as indicated. Results are representative of 3 independent analyses.

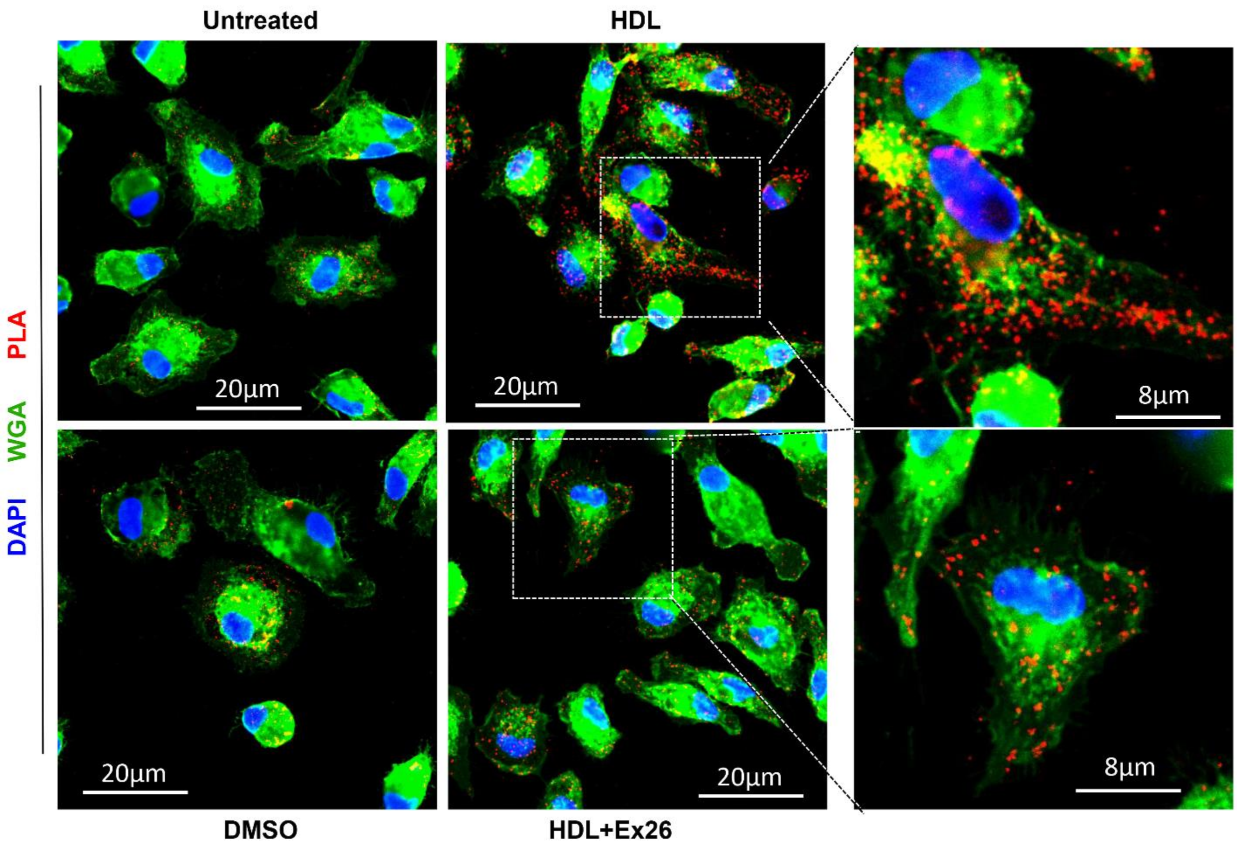

Supplementary Figure 2: Co-staining of macrophages for SR-B1/S1PR1-GFP PLA and WGA. Thioglycollate-elicited peritoneal macrophages from *S1pr1*$^{eGFP/eGFP}$ mice were treated with or without HDL and with Ex26 (10 µM added as a stock in DMSO) or with DMSO as a vehicle control, as indicated. After 30 min, cells were fixed and incubated with Alexa-488-labeled WGA (green fluorescence). Cells were then permeabilized and PLA analysis using antibodies against SR-B1 and the GFP tag of S1PR1 (red fluorescence). Images were captured at 63 × using a Stellaris 5 Confocal Microscope from Leica Microsystems. Images on the right correspond to zoomed in views of the boxed areas in the images in the middle (HDL treated without or with Ex26).

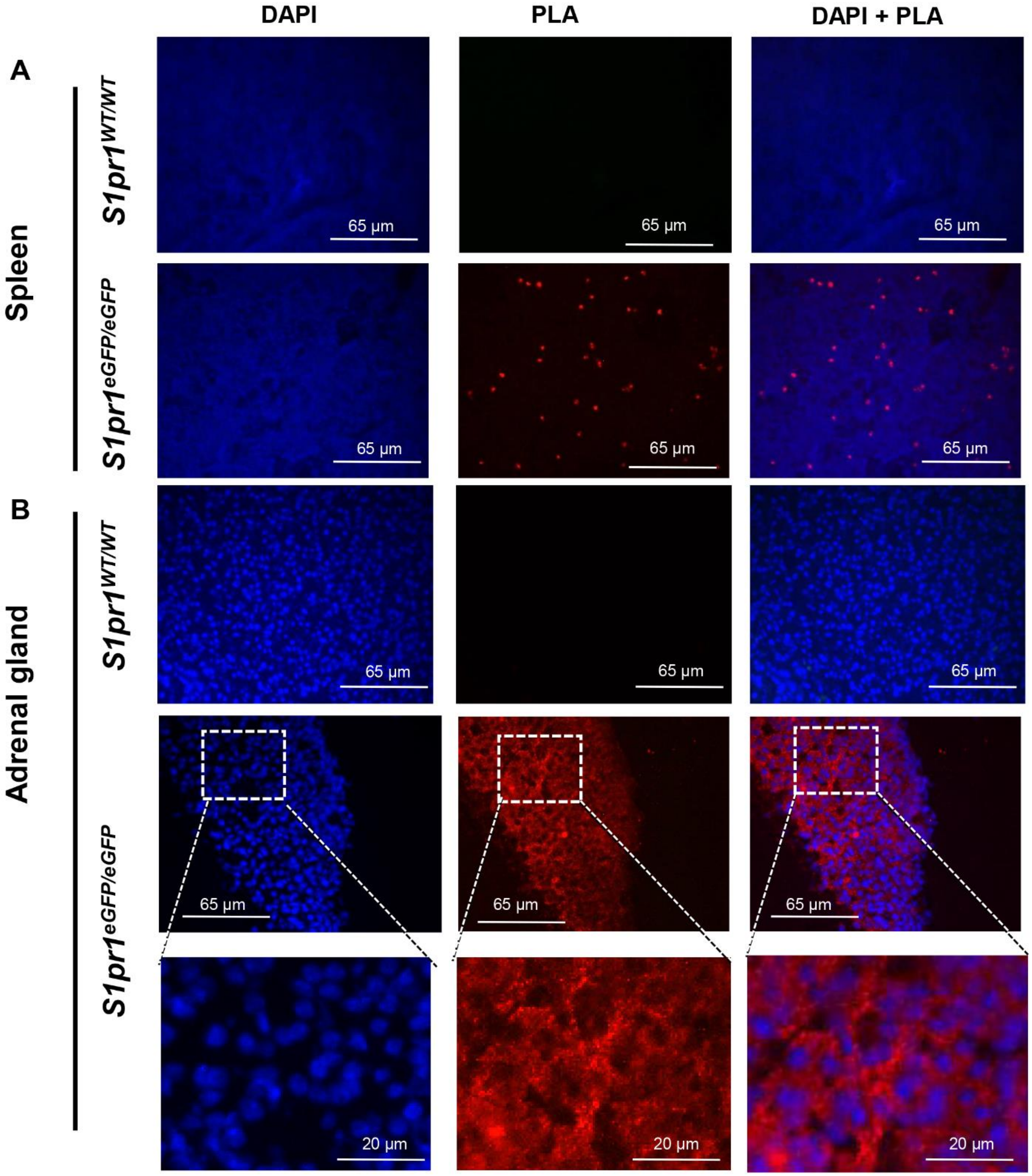

Supplementary Figure 3: SR-B1 and S1PR1 interactions detected in spleens and adrenal glands of *S1pr1*$^{eGFP/eGFP}$ mice. (A) Spleens and (B) adrenal glands from *S1pr1*$^{WT/WT}$ (C57BL/6J) and *S1pr1*$^{eGFP/eGFP}$ mice were collected and cryosectioned. PLA analysis for SR-B1 and S1PR1-GFP interactions was carried out (red fluorescence) followed by DAPI staining for nuclei (blue fluorescence). Images were captured at 40× using a Stellaris 5 Confocal Microscope from Leica Microsystems. Images in the bottom row correspond to zoomed-in views of the boxed areas in the images of the *S1pr1*$^{eGFP/eGFP}$ adrenal gland.
